# Antimicrobial Resistance and Hospital- and Community-Associated Infections

**DOI:** 10.3390/antibiotics14050514

**Published:** 2025-05-16

**Authors:** Samantha Flores-Treviño

**Affiliations:** Department of Infectious Diseases, Hospital Universitario Dr. José Eleuterio González, Universidad Autónoma de Nuevo León, Monterrey 64460, Mexico; samantha.florestr@uanl.edu.mx

Antimicrobial resistance (AMR) poses a significant global threat to human health, and was estimated to be associated with almost one million deaths in 2019 [1,2]. The World Health Organization (WHO) recently issued an updated *Bacterial Priority Pathogens List* including antimicrobial-resistant microorganisms [3]. Among Gram-negative bacteria resistant to last-resort antibiotics, studies on *Acinetobacter baumannii* and the Enterobacterales order are critical due to their ability to transfer resistance genes. In addition, studies on antimicrobial-resistant *Pseudomonas aeruginosa*, *Staphylococcus aureus* and *Enterococcus faecium* are of high priority due to the threat they pose in healthcare settings. Healthcare-associated infections (HAIs) caused by ESKAPE pathogens (vancomycin-resistant *Enterococcus* spp. [VRE], methicillin-resistant *S. aureus* [MRSA], carbapenem-resistant *Klebsiella pneumoniae*, multidrug-resistant [MDR] *A. baumannii*, MDR *P. aeruginosa*, and carbapenem-resistant *Enterobacter* spp.) are known to cause high morbidity and mortality in patients due to their acquired resistance to last-resort antibiotics [4,5,6]. Patients in intensive care units or those who are immunocompromised are most affected by HAIs caused by MDR pathogens, which can increase disease severity. The most frequent HAIs are bloodstream infections, ventilator-associated pneumonia and surgical site infections [7].

The contributions to this Special Issue regarding Gram-negative pathogens include the following: Luna-De-Alba et al. (2024) conducted an assessment of different antimicrobial combinations against MDR or extensive drug-resistant (XDR) *A. baumannii* strains. Most strains carried carbapenemase OXA-24/40, aminoglycoside-modifying enzymes, and *parC* gene mutations; overexpressed AdeIJK, AdeABC, and AdeFGH efflux pumps and CarO membrane porin; and under-expressed Omp33-36, OmpA, and CarO membrane porins, and showed low biofilm production. Interestingly, antimicrobial combinations such as levofloxacin-ampicillin/sulbactam and meropenem-colistin inhibited bacterial growth. In the contribution by Papa-Ezdra et al. (2024), MDR *P. aeruginosa* strains that caused an outbreak in an ICU were analyzed and found to all be clonally related and belong to ST309, an emerging high-risk clone in the Americas. The strains were resistant to ceftazidime, cefepime, amikacin, and ceftolozane-tazobactam, and harbored *bla*_PER-1_ and *qnrVC* genes. Yamaki et al. (2024) assessed the clinical outcomes of antibiotic modifications in patients with infections caused by Gram-negative pathogens exhibiting resistance to extended-spectrum cephalosporins. Although 72% of patients had antibiotic regimen modifications, their clinical outcomes showed no differences, which highlights the importance of identifying patients at risk for resistant organisms early in admission.

In the contribution by Chotiprasitsakul et al. (2023), an assessment of the epidemiology of community-onset bloodstream infections in Thailand showed that 25% of infections were antimicrobial-resistant AMR, and one-third of Enterobacterales (*Escherichia coli* and *K. pneumoniae*) were not susceptible to ceftriaxone. Li et al. (2023) assessed the effectiveness of multi-model strategies on HAIs caused by MDR pathogens in rehabilitation units in China, which decreased the burden of HAIs in general and of HAIs caused by MDR pathogens, in addition to the contamination rate of MDR pathogens in the environmental setting.

Kumar et al. (2024) overviewed the epidemiology of MDR sepsis and found that underlying comorbidities, old age, antibiotic overuse and inadequate empiric therapy contribute to recurrent sepsis, with high mortality rates. Effective sepsis treatment includes the use of antimicrobial combination therapy and the exploitation of local pathogen resistance patterns.

The contributions to this Special Issue regarding Gram-positive pathogens include the following: Balasiu and MacKenzie (2023) conducted an assessment of teicoplanin resistance in coagulase-negative staphylococci (CoNS), which was challenging and influenced by technical factors. Their results emphasized the need for future studies focusing on the clinical efficacy of teicoplanin in relation to its susceptibility. Sohail et al. (2023) assessed MRSA isolates from Pakistan during the COVID-19 pandemic, of which 56% were HAIs and 44% were community-acquired. Most MRSA isolates detected were weak biofilm producers and adhesion genes (*clfB*, *icaAD*, *fib*, *sdrC*, *eno*, *fnbA*, *sdrE*, *icaBC*, *clfA*, *fnbB*, *sdrD*, and *cna*). In the contribution by Worku et al. (2023), the prevalence of MRSA was evaluated in surgical site infections in Ethiopia. Patients positive for *S. aureus* accounted for 21.6% of all patients, among which 24.5% had MRSA; moreover, the *mecA* gene was detected in 27.5% of isolates. Among the risk factors associated with MRSA infections were older age, prolonged hospitalization and previous antibiotic administration.

*Clostridioides difficile* is one of the most common pathogens in hospitalized patients receiving antimicrobial therapy and is the leading cause of hospitalization [8]. Salas-Treviño et al. (2025) showed that the co-colonization of *C. difficile* and other non-*difficile* Clostridia (*Clostridium ramosum* or *Clostridium innocuum*) in patients with antibiotic-associated diarrhea was correlated with treatment extension and failure.

The main conclusion drawn from the above contributions is that AMR is still an issue in most hospitals worldwide; thus, hospitals should strengthen their strategies for infection prevention, continue surveillance of antimicrobial resistance genes, and promote the implementation of antimicrobial stewardship programs. More prevention and control strategies are needed to reduce the burden of antimicrobial resistance in both hospitals and communities.
